# Induction of Intrahepatic HCV NS4B, NS5A and NS5B-Specific Cellular Immune Responses following Peripheral Immunization

**DOI:** 10.1371/journal.pone.0052165

**Published:** 2012-12-21

**Authors:** Krystle A. Lang Kuhs, Roberta Toporovski, Arielle A. Ginsberg, Devon J. Shedlock, David B. Weiner

**Affiliations:** 1 Laboratory Medicine, Department of Pathology, University of Pennsylvania, Philadelphia, Pennsylvania, United States of America; University of Washington, United States of America

## Abstract

Numerous studies have suggested that an effective Hepatitis C Virus (HCV) vaccine must induce strong cytotoxic and IFN-γ+ T cell responses targeting the non-structural region of the virus. Most importantly, these responses must be able to migrate into and remain functional within the liver, an organ known to cause T cell tolerance. Using three novel HCV DNA vaccines encoding non-structural proteins NS4B, NS5A and NS5B, we assessed the ability of peripheral immunization to induce functional intrahepatic immunity both in the presence and absence of cognate HCV antigen expression within the liver. We have shown that these constructs induced potent HCV-specific CD4+ and CD8+ T cell responses in the spleen of C57BL/6 mice and that these responses were detected within the liver following peripheral immunization. Additionally, using a transfection method to express HCV antigen within the liver, we showed that intrahepatic HCV-specific T cells remained highly functional within the liver and retained the ability to become highly activated as evidenced by upregulation of IFN-γ and clearance of HCV protein expressing hepatocytes. Taken together, these findings suggest that peripheral immunization can induce potent HCV-specific T cell responses able to traffic to and function within the tolerant environment of the liver.

## Introduction

Perhaps the greatest challenge in vaccine development for Hepatitis C Virus is that unlike other hepatitis viruses, such as Hepatitis A and Hepatitis B, where successful antibody-based vaccines have been created, protection against HCV infection does not appear to be antibody mediated [1], [2]. Instead, numerous studies in both chimpanzees and acutely infected humans have supported the idea that an effective HCV vaccine must be able to induce strong HCV-specific cytotoxic IFN-γ+ T cells able to target the non-structural region of the virus and that most importantly, these cells must be able to migrate to and become activated within the liver, an organ known to induce T cell tolerance [3]–[7].

While it is known that strong intrahepatic HCV-specific T cell responses are correlated with clearance of acute infection, most HCV infected individuals fail to either mount or sustain these responses resulting in the progression to chronic infection [7]. Part of this failure has been attributed to the tolerant immunological environment of the liver and its ability to negatively modulate T cell responses [8]. Constantly inundated with both gut and bacterial antigens, the liver is believed to play an important role in tolerizing T cells to food and gut antigens [9], [10]. The liver is thought to induce tolerance by maintaining an environment of abundant IL-10 production [8] and low expression of co-stimulatory molecules [11], [12]. This immunmodulatory environment has been known to cause dysfunction and apoptosis of CD8+ T cells which, has been associated with liver-induced T cell tolerance to food, transplant and hepatotropic viral antigens [13]–[16].

Therefore, an effective T cell based HCV vaccine must be able to induce functional HCV-specific T cell responses within the immune modulatory environment of the liver. Previous studies from our laboratory and others have shown that vaccines targeting the NS3 region of the virus are able to induce functional T cell responses in the liver [17]–[19]. However, to date there have been no studies which have specifically characterized the ability of other HCV proteins within the non-structural region of the virus, such as NS4B, NS5A and NS5B, to elicit functional T cell responses within the liver. Given the high mutational rate of the virus, it is essential for an effective HCV vaccine to induce functional intrahepatic T cell responses able to target multiple epitopes within the non-structural region of the virus. Therefore, in the present study, we asked whether peripheral immunization could induce HCV NS4B, NS5A and NS5B-specific intrahepatic T cell responses and if so, whether these HCV-specific T cells were able to become activated and functional upon liver expression of their cognate antigen. To answer these questions, we took a multi-step approach to design three different DNA vaccines able to induce potent cytotoxic and IFN-γ+ HCV-specific T cell responses directed against the non-structural proteins, NS4B, NS5A and NS5B. We show that these constructs, pConNS4B, pConNS5A and pConNS5B are expressed *in vitro* and are able to induce strong HCV-specific T cell responses in the spleen. Additionally, we have shown that HCV-specific T cells induced through peripheral immunization are recruited into the resting liver and retain the ability to become highly activated following liver expression of their cognate antigen, resulting in increased IFN-γ expression and clearance of HCV antigen expressing hepatocytes.

## Materials and Methods

### Ethics Statement

The animals used in this study were maintained in accordance with the National Institutes of Health and the University of Pennsylvania Institutional Care and Use Committee (IACUC) guidelines. The protocol for this study was approved by the University of Pennsylvania's Animal Care and Use Committee (Permit Number: 801244).

### Generation of pConNS4B, pConNS5A, pConNS5B

The consensus sequences for NS4B and NS5B were generated from 174 different genotype 1a sequences and the consensus sequence for NS5B was generated from 259 genotype 1b sequences obtained from Los Alamos National Laboratory HCV Sequence Database (**Table S1**). The Los Alamos National Laboratory “Consensus” tool (http://hcv.lanl.gov/content/sequence/HCV/ToolsOutline.html) was used to align the sequences and to generate the three final consensus sequences. In order to inhibit the activity of the consensus proteins *in vivo*, several mutations were made. For NS4B a C261T mutation was made to inhibit polymerization by interfering with protein-protein interactions and preventing the formation of replication complex [20]. For NS5A three mutations were made; a C59G mutation to interfere with the conserved zinc finger domain preventing viral replication [21] and; T213A and K215G, mutations that have been shown to stop binding of hVAP-A, which is important for viral replication [22]. For NS5B, a Y276A mutation was made which has been shown to prevent RNA polymerase activity RNA template/primer association [23].

An N-term IgE leader sequence and a C-term HA tag were added to each construct. The sequences were codon and RNA optimized using GeneOptimizer™ (GENEART, Germany). The final consensus sequences were synthesized, sequence verified and inserted in to the clinical expression vector pVAX (Invitrogen) by GENEART (Germany). The final constructs were named pConNS4B, pConNS5A and pConNS5B. The final DNA and protein sequences of pConNS4B, pConNS5A and pConNS5B can be found in **Figures S1**, **S2** and **S3**, respectively.

### Immunofluorescence

RD muscle cells were transfected with pConNS4B, pConNS5A and pConNS5B using Lipofectamine™ (Invitrogen) according to the manufacturer's guidelines for 48 hours. Expression of each protein was detected with an anti-HA polyclonal rabbit antibody (Invitrogen) followed by a Cy3 conjugated goat anti-rabbit secondary antibody (Invitrogen).

### Immunization/Electroporation

Six to eight week old female C57BL/6 mice were purchased from Jackson Laboratories and were maintained in accordance with the National Institutes of Health and the University of Pennsylvania Institutional Care and Use Committee (IACUC) guidelines. The protocol for this study was approved by the University of Pennsylvania's Animal Care and Use Committee (Permit Number: 801244).

Five mice were used per group. Each mouse received a total of two immunizations, two weeks apart and was sacrificed one week following the second immunization. Immunizations were given intramuscularly, followed by electroporation with the CELLECTRA® adaptive constant current electroporation device and electrode arrays. The final dosing for the constructs was as follows; pConNS4B, 12.5 µg; pConNS5A, 5 µg and pConNS5B, 12.5 µg.

### Splenocyte Isolation

Spleens were isolated and individually crushed with the use of a Stomacher device. Splenocytes were strained with a 40 µM cell strainer and treated 5 min with ACK lysis buffer (Biosource) to lyse RBCs. The splenocytes were resuspended in complete media (RPMI 1640 with 2 mM/L L-glutamine supplemented with 10% heat inactivated FBS, 1× anti-biotic/anti-mycotic, and 55 µM/L β-mercaptoethanol).

### Liver Lymphocyte Isolation

Livers were individually pulverized using a Stomacher machine, strained and treated 5 min with 10 ml ACK lysis buffer (Bioscience). The mixture was pelleted and the hepatocytes were separated from the lymphocytes through the use of a 35% percoll (GE Healthcare) gradient. The pelleted lymphocytes were resuspended in complete media. Experiments were performed with and without liver perfusion and no differences were observed.

### IFN-γ ELISpot

The mouse IFN-γ ELISpot assays were conducted as previously described [24]. The splenocytes were stimulated with pools of 15mer peptides over lapping by 8 amino acids and spanning the length of each construct (**Table S2**). Peptides were synthesized by Genscript (Piscataway, NJ), resuspended in DMSO and pooled at a concentration of 2 µg/ml/peptide. The splenocytes were plated at a concentration of 200,000 cells per well. Results were adjusted and graphed as the average number of spot forming units (SFU) per 1×10^6^ splenocytes.

### Flow Cytometry Reagents

The following directly conjugated antibodies were used: anti-mouse CD3- allophycocyanin cyanine dye 7 (APC-Cy7) [clone 145-C11], anti-mouse CD4- fluorescein isothiocyanate (FITC) [clone H129.19], anti-mouse CD8- peridinin chlorophyll protein 5.5 (PerCP5.5) [clone 53-6.7], anti-mouse IFN-γ- phycoerythryin cyanine dye 7 (PE-Cy7) [clone XMG1.2] (all from BD Biosciences, San Jose, CA). Aqua Live/Dead fixable dead cell Stain Kit (Molecular Probes, Eugene, OR) was used according to manufacturer's protocol to identify live cells.

Samples were collected on a LSRII flow cytometer (BD Biosciences, Franklin Lakes, NJ). Data was analyzed using FlowJo software, version 8.7.1 for Mac, (Tree Star, Ashland, OR).

### Intracellular Cytokine Staining

Splenocytes were resuspended in complete media at a concentration of 1×10^6^ cells/100µl and plated in a round bottom 96-well plate. Splenocytes were stimulated with 100 µl of: 1) 2 µg/ml pConNS4B, pConNS5A or pConNS5B overlapping peptides, 2) 1 µg/ml Staphylococcus enterotoxin B (positive control; Sigma-Aldrich, St. Louis, MO) or 3) 0.1% dimethyl sulfoxide (negative control) all diluted in complete media supplemented with GolgiStop and GolgiPlug (BD Bioscience). Splenocytes were stimulated for 5 hours at 37°C, washed with PBS and stained for viability. Splenocytes were stained extracellularly for; anti- CD4, CD8 for 30 min at 4°C. Splenocytes were permeabilized and washed using BD Cytofix/Cytoperm Solution Kit (BD Bioscience) and stained intracellularly with anti- IFN-γ and CD3 for 45 min at 4°C. Splenocytes were stored at 4°C until analysis. Specific function was reported as the percent function of the peptide stimulated group minus the percent function of the 0.1% dimethyl sulfoxide stimulated group (negative control).

### Confocal Microscopy

Liver biopsies were fixed in 2% paraformaldehyde followed by overnight cryoprotection in 30% sucrose. Biopsies were quick frozen in Tissue-Tek OCT (Bayer Corporation, Pittsburgh, PA). Tissue sections (6 µm) were mounted on Superfrost Plus glass slides (Fisher Scientific, Pittsburgh, PA) and kept at 80°C until use. For staining slides were brought to room temperature (RT), washed (three 10 min washes in PBS), and blocked in PBS containing 10% serum and 0.1% Triton. Sections were incubated for 1 hour at RT or overnight at 4°C in primary reagents, washed and secondary reagents were applied for 30 min at RT. The sections were counterstained with DAPI (Invitrogen, Carlsbad, CA). Coverslips were mounted with Prolong Gold mounting media (Invitrogen, Carlsbad, CA). Monoclonal antibodies used were mouse anti-HA.11 (clone 16B12; Covance, Emeryville, CA). All images were obtained using a Zeiss Axiovert 100 inverted confocal microscope and Image J software (NIH, Rockville, MD) was used for analysis. For each group, three random images were captured for each animal (n = 5) by a microscopist blinded to the vaccine status of the animals. MFI values for NS4B, NS5A or NS5B (red) were calculated and normalized to the MFI value for DAPI (blue) for each image.

### Liver transfection

Animals were tail vein injected with 100 µg of pConNS4B, pConNS5A or pConNS5B diluted in 300 µl of PBS. Animals were sacrificed 48 hours following injection. This method for inducing expression of foreign antigen within the liver has been previously described [25].

## Results

### Design, expression and immunogenicity of pConNS4B, pConNS5A and pConNS5B

**Figure 1:**
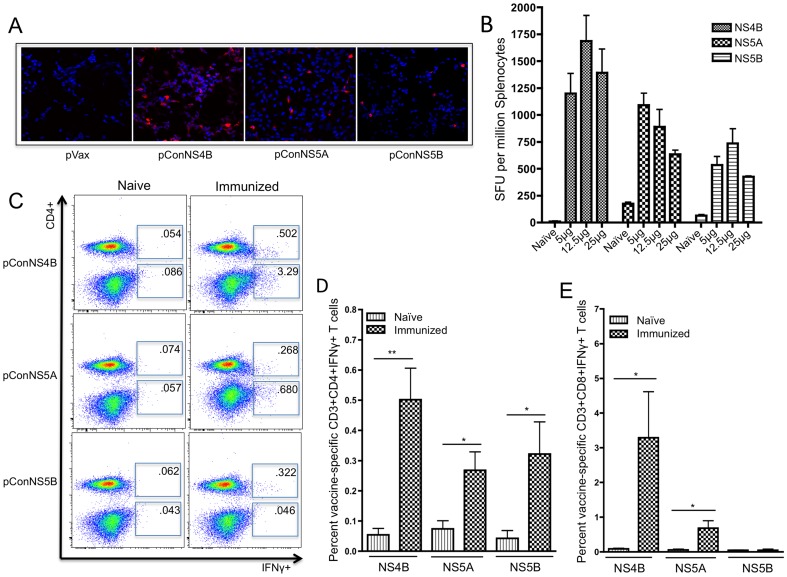
Expression and immunogenicity of pConNS4B, pConNS5A and pConNS5B. **A**) Human RD muscle cells were transiently transfected with each construct. Expression of each gene product was detected using an anti-HA monoclonal antibody and confocal microscopy (250×). **B**) IFN-γ ELISpot dose response. Animals (n = 5 per group) were immunized with 5 µg, 12.5 µg or 25 µg of pConNS4B, pConNS5A and pConNS5B. Animals received a total of two intramuscular immunizations, two weeks apart followed by electroporation. Animals were sacrificed one week following the last immunization after which splenocytes were isolated and analyzed. The response of each animal to each dose was determined with IFN-γ ELISpot assays. Splenocytes were isolated and individually analyzed for NS4B-, NS5A- or NS5B-specific T cell responses. **C, D, E**) Splenocytes were intracellularly stained for IFN-γ and analyzed with flow cytometry. **C**) Representative animal from each group. The values shown are the averaged response of five individual animals from each group. **D**) and **E**) graphical representation of percent HCV-specific IFN-γ+ T cell responses from isolated splenocytes. Values are reported as the average percent ± SE of the **D**) CD4+ IFN-γ+ or **E**) CD8+ IFN-γ+ T cell responses of each animal (n = 5) from each group. Significance was determined by Student's *t* test (*p<0.05, **p<0.005 and ***p<0.0005).

Due to its ability to rapidly mutate, HCV is highly variable in sequence and is currently classified into 6 different genotypes with more than 50 subtypes. Genotype 1 is the most prevalent in North America and Europe and is by far the most difficult to treat. It is estimated that genotype 1 accounts for 73 percent of all HCV infections in the US, with genotype 1a and 1b accounting for up to 51.6 and 26.5 percent of all cases, respectively [26]. Therefore, when designing our constructs we focused our efforts on HCV genotypes 1a and 1b. In order to account for the high genetic diversity of the virus, we decided to take a consensus immunogen approach. Recent studies conducted in both our laboratory and others have suggested that consensus antigens may enhance immune responses by increasing the breadth of cellular immune responses when compared to native antigens [24], [27], [28]. Therefore, due to the high mutational rate of the virus, it is believed that HCV vaccines able to elicit broader immune responses may be better able to control infection and prevent escape mutants. Given that genotype 1a accounts for the majority of genotype 1 infections, we designed two genotype 1a constructs (pConNS4B and pConNS5B) and one genotype 1b construct (pConNS5A). Protein expression of each construct was confirmed through transfection of human RD muscle cells *in vitro*. An anti-HA polyclonal antibody was used to detect expression of the constructs 48 hours following transfection. The cells were visualized using confocal microscopy at 250× magnification. All three constructs were expressed as evidenced by specific staining of construct-transfected cells. Transfection with the empty vector pVax was used as a control, **Fig. 1A**.

Once expression was confirmed, the cellular immunogenicity of the constructs was determined. Animals were separated into three dosing groups for each construct with five animals per group. The animals were immunized intramuscularly with 5 µg, 12.5 µg or 25 µg of pConNS4B, pConNS5A or pConNS5B, followed by electroporation. The animals received two immunizations, two weeks apart and were sacrificed one week following the second immunization. Immunogenicity was determined by IFN-γ ELISpot assays, the results of which can be seen in **Fig. 1B**. While all constructs were strongly immunogenic, responses for pConNS4B were the largest while responses for pConNS5B were the lowest. The optimum dose for pConNS4B was 12.5 µg (1687±237 SFU/10^6^ splenocytes), for pConNS5A was 5 µg (1091±111 SFU/10^6^ splenocytes) and for pConNS5B was 12.5 µg (736±136 SFU/10^6^ splenocytes), although the differences between doses were small.

### Immunization induced potent NS4B-, NS5A- and NS5B- specific CD4+ and CD8+ T cells within the spleen

Once the dosing was determined, a more detailed analysis of the cellular immune responses was performed. Dosing for the constructs in all subsequent experiments was as follows; pConNS4B, 12.5 µg; pConNS5A, 5 µg and pConNS5B, 12.5 µg. In order to determine the relative contributions of CD8+ and CD4+ T cell responses for each construct, splenocytes were intracellularly stained for IFN-γ and visualized with flow cytometry, **Fig. 1C**. Results of the intracellular cytokine staining correlated well with results of the IFN-γ ELISpot assays. The majority of the IFN-γ responses to pConNS4B and pConNS5A were produced by CD8+ T cells, although CD4+ T cells specific for each construct were also identified. Interestingly, the majority of the IFN-γ response to pConNS5B was CD4+ T cell mediated, with few IFN-γ+ CD8+ T cells identified. The average percentage of IFN-γ+ CD4+ T cells for pConNS4B, pConNS5A and pConNS5B were 0.50%±0.11%, 0.27%±0.06% and 0.32%±0.11%, respectively, **Fig. 1D**. The average percentage of IFN-γ+ CD8+ T cells for pConNS4B and pConNS5A were 3.29%±1.33% and 0.68%±0.22%, respectively, **Fig. 1E**.

### Immunization induced potent NS4B-, NS5A- and NS5B- specific CD4+ and CD8+ T cells within the liver

**Figure 2:**
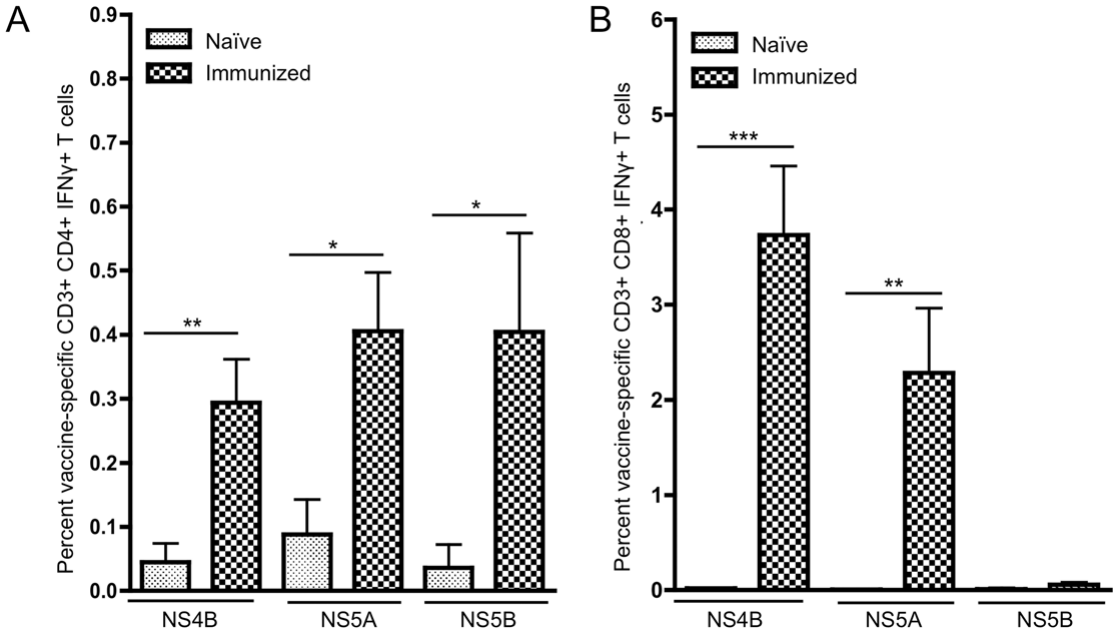
Flow cytometric analysis of the percentage of HCV-specific IFN-γ+ T cell responses from isolated liver lymphocytes. Values are reported as the average percent ± SE of HCV-specific **A**) CD4+ IFN-γ+ or **B**) CD8+ IFN-γ+ T cell responses of each animal (n = 5) from each group. Significance was determined by Student's *t* test (*p<0.05, **p<0.005 and ***p<0.0005).

Given the importance of intrahepatic T cell responses in the clearance of HCV infection, once we had determined that our constructs were able to induce strong T cell responses in the periphery, we wanted to determine whether these responses could be detected within the liver. Mice were immunized as previously described. Liver lymphocytes were isolated from each animal and HCV-specific T cells were identified by IFN-γ expression and flow cytometry. Interestingly, HCV-specific T cells were identified in the livers of all immunized mice, **Fig. 2**. Both CD4+ and CD8+ T cell responses were detected within the livers of mice immunized with pConNS4B and pConNS5A, with only CD4+ T cell responses detected in mice immunized with pConNS5B. The dominant T cell responses detected within the liver were the same as those identified within the spleen. Mice immunized with pConNS4B and pConNS5A had strong CD8+ T cell responses within the liver, while mice immunized with pConNS5B showed mainly CD4+ T cell responses and few CD8+ T cell responses. The CD4+ T cell responses for pConNS4B, pConNS5A and pConNS5B were 0.29%±0.07%, 0.41%±0.09% and 0.41%±0.06%, respectively, **Fig. 2A**. The CD8+ T cell responses for pConNS4B, pConNS5A and pConNS5B were 3.73%±0.73%, 2.28%±0.68% and 0.06%±0.02%, respectively, **Fig. 2B**.

### Liver expression of HCV antigens resulted in increased IFN-γ production and clearance of transfected hepatocytes

**Figure 3:**
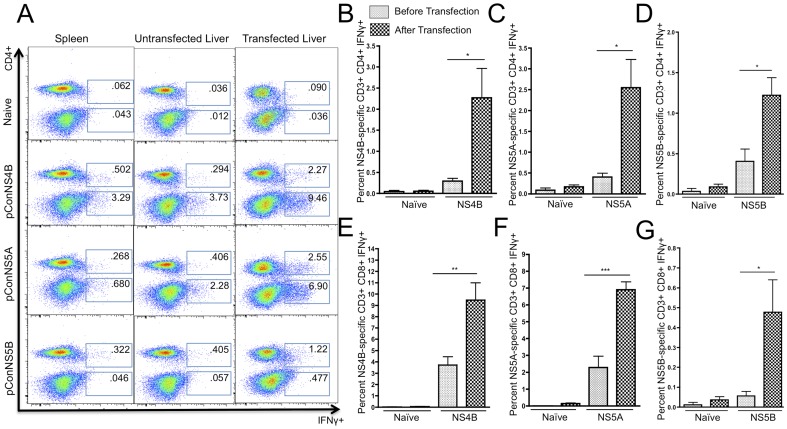
Flow cytometric analysis of the percentage of HCV-specific IFN-γ+ T cell responses from isolated lymphocytes from the spleen, untransfected liver and transfected liver. Lymphocytes from each animal (n = 5) were isolated and individually analyzed for NS4B-, NS5A- or NS5B-specific T cell responses. The isolated lymphocytes were intracellularly stained for IFN-γ and analyzed with flow cytometry. **A**) Representative animal from each group. The values shown are the averaged response of five individual animals. **B**), **C**) and **D**) Graphical representation comparing the average percentage of HCV-specific CD4+ IFN-γ+ T cell responses in the liver before and after transfection. Values are reported as the average percent ± SE of CD4+ IFN-γ+ T cell responses to **A**) pConNS4B, **B**) pConNS5A, **C**) pConNS5B. **E, F,** and **G**) Graphical representation comparing the average percentage of HCV-specific CD8+ IFN-γ+ T cell responses in the liver before and after transfection. Values are reported as the average percent ± SE of CD8+ IFN-γ+ T cell responses to **E**) pConNS4B, **F**) pConNS5A, **G**) pConNS5B. Significance was determined by Student's *t* test (*p<0.05, **p<0.005 and ***p<0.0005).

Next, we sought to determine whether liver-specific expression of either NS4B, NS5A or NS5B proteins could activate the HCV-specific T cells detected within the liver. In order to induce liver expression of NS4B, NS5A and NS5B, the hepatocytes of immunized mice were transfected by administering a tail vein injection of pConNS4B, pConNS5A or pConNS5B. This method for inducing expression of foreign antigen within the liver has been previously described [25]. The livers were allowed to transfect for 48 hours, after which they were harvested and the liver lymphocytes were isolated. As mentioned before, immunization induced HCV-specific T cells were identified by IFN-γ secretion detected through intracellular cytokine staining and flow cytometry. Following the tail vein injections, a massive increase in the percentage of both CD4+ and CD8+ HCV-specific T cells was seen in all three immunization groups as compared to both the spleen and the untransfected liver, **Fig. 3A**. The percentage of CD4+ HCV-specific T cells was 2.27%±0.70%, 2.55%±0.70% and 1.22%±0.22% for mice immunized with pConNS4B, pConNS5A and pConNS5B, respectively, **Fig. 3B, C, D**. The percentage of CD8+ HCV-specific T cells was 9.46%±1.53%, 6.98%±0.48% and 0.477%±0.16% for mice immunized with pConNS4B, pConNS5A and pConNS5B, respectively, **Fig. 3 E, F, G**. The largest fold increase, as determined by the percentage of HCV-specific IFN-γ+ T cells in the liver before and after tail vein injection, was seen with the CD4+ T cell response. The fold increase in the intrahepatic CD4+ T cell response in pConNS4B, pConNS5A and pConNS5B immunized mice was approximately 8, 6 and 3 fold, respectively. While the CD8+ T cell response remained the dominant response in the liver both before and after tail vein injection, a slightly smaller fold increase was seen with the CD8+ T cells response as compared to the CD4+ T cells response. The fold increase in the intrahepatic CD8+ T cell response in pConNS4B, pConNS5A and pConNS5B immunized mice was approximately 3, 3 and 8 fold, respectively.

**Figure 4:**
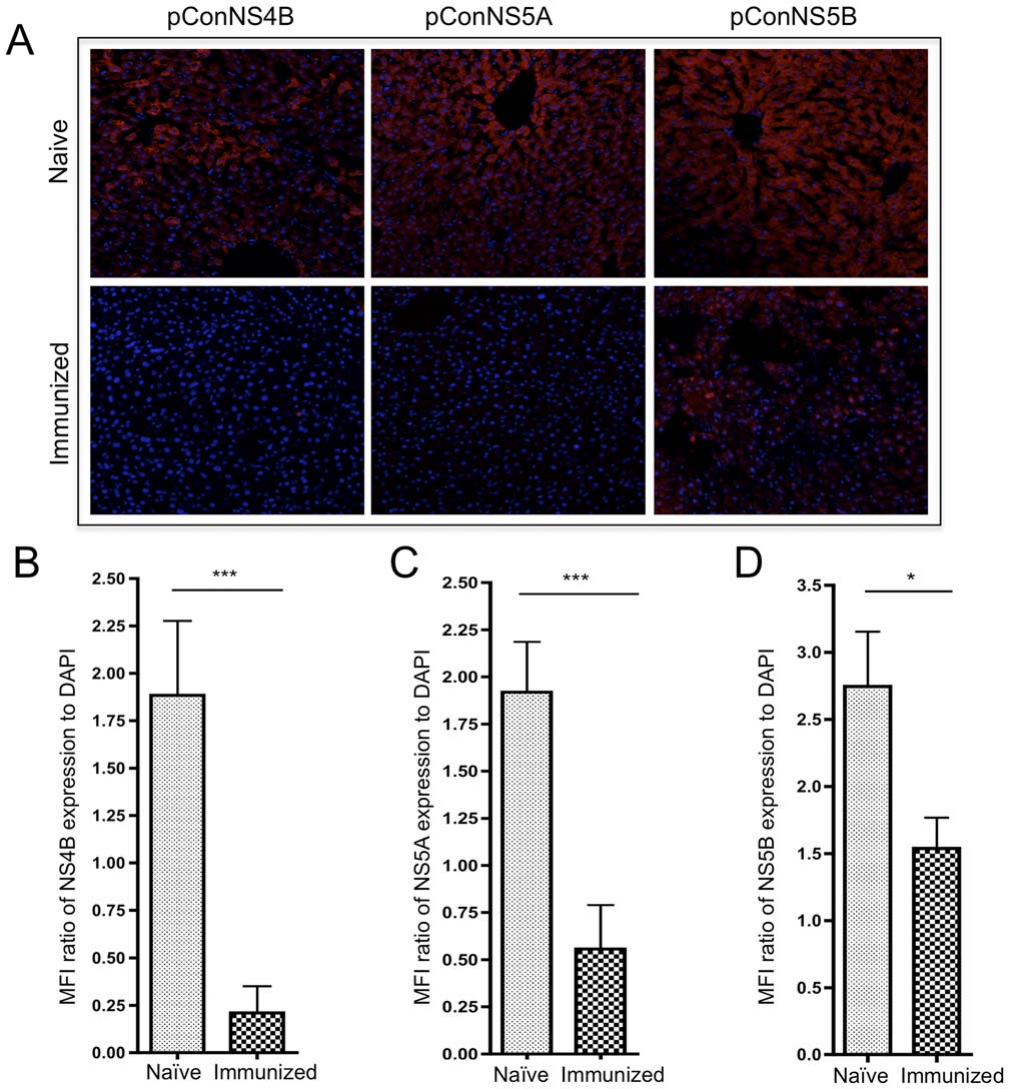
Clearance of NS4B, NS5A or NS5B transfected hepatocytes. **A**) Confocal microscopy. Representative confocal image of hepatocyte expression of NS4B, NS5A or NS5B in each group. Expression of each construct was detected with an anti-HA antibody (red). Nuclei were stained with DAPI (blue). **B, C** and **D**) Graph of MFI ratio of expression of **B**) NS4B, **C**) NS5A or **D**) NS5B as normalized to DAPI. For each group, three images were captured for each animal (n = 5). MFI values for NS4B, NS5A or NS5B (red) were calculated and normalized to the MFI value for DAPI (blue) for each image. The values shown are the averaged response ± SE of five individual animals from both the naïve and immunized groups. Significance was determined by Student's *t* test (*p<0.05, **p<0.005 and ***p<0.0005).

Next, we determined whether this response was able to clear HCV protein expressing hepatocytes. To test this, a lobe of liver from each animal was stained for expression of NS4B, NS5A or NS5B. Clearance was assessed by measuring the expression of NS4B, NS5A or NS5B in hepatocytes of immunized mice versus naïve controls. Representative confocal images for each group are shown in **Fig. 4A**. Clearance of transfected hepatocytes for each group was quantified by the mean florescent intensity (MFI) of NS4B, NS5A or NS5B expression normalized to the number of cells present within each field as measured by the MFI of nuclear DAPI staining, **Fig. 4B, C and D**. Compared to the naïve controls, dramatic reductions in the number of transfected hepatocytes were seen in the all three immunization groups. Animals immunized with pConNS4B, pConNS5A or pConNS5B had approximately 9, 3 and 2 fold reductions in foreign antigen expression within the liver compared to naïve controls. The amount of clearance observed in each immunization group correlated well with the HCV-specific CD8+ T cell response detected within the transfected livers.

## Discussion

Numerous studies support the idea that a successful HCV vaccine must be able to induce strong HCV-specific T cell responses able to target the non-structural region and that these cells must be able to function within the liver [3]–[7]. While there is strong agreement on the type of responses needed for an effective HCV vaccine, to date there has been little research aimed at elucidating whether vaccines targeting non-structural proteins other than NS3 can induce potent T cell responses within the liver. In this present study, we were able to show that each vaccine construct expressed its respective protein and furthermore immune analysis demonstrated that all three constructs were able to induce potent CD8+ and CD4+ IFN-γ+ HCV-specific T cells in the periphery following only two immunizations. In fact, immunization with pConNS4B produced such strong cellular immune responses that more than 3 percent of all CD8+ T cells isolated from the spleen where NS4B-specific.

However, the most important question was whether peripheral immunization with pConNS4B, pConNS5A and pConNS5B could result in functional HCV-specific immunity within the liver. Following immunization, not only were NS4B-, NS5A- and NS5B- specific CD4+ and CD8+ T cell responses detected in the liver following peripheral immunization, but these cells were found at similar percentages within the liver as observed in the spleen suggesting that peripheral immunization with these constructs was in fact able to result in a large pool of vaccine-specific T cells within the liver. Likewise, this pool of vaccine-specific T cells was shown to be fully functional as evidenced by IFN-γ production in flow cytometric analysis.

However, since it has been reported that the liver has the ability to specifically delete activated T cells [11], [12], we were interested in how HCV-specific T cells induced through vaccination with our constructs would function when activated in the liver following intrahepatic expression of their respective cognate antigens. Following hepatocyte expression, not only were HCV-specific T cells isolated from the livers of immunized mice, but these T cells had the ability to become highly activated within the liver as evidenced by the large percent increase in IFN-γ+ CD4+ and CD8+ T cells and the rapid clearance of HCV transfected hepatocytes. In fact, responses in the liver were so potent that animals immunized with pConNS4B or pConNS5A had very few if any observable transfected hepatocytes and likewise, a substantial decrease in the number of transfected hepatocytes among animals immunized with pConNS5B was also observed despite the much lower frequency of detectable vaccine-specific T cells compared to the other vaccine groups.

Therefore, our study is to the first to suggest that peripheral immunization targeting the HCV non-structural proteins NS4B, NS5A and NS5B can result in the formation of a large pool HCV-specific T cells within the liver. Likewise, we have shown that this pool of HCV-specific T cells remains fully functional within the liver. Given that it has been previously reported that T cell infiltration into the liver is not observed until 72 hours following liver transfection [19], the rapid clearance of HCV transfected hepatocytes within only 48 hours appears to be critically dependent on the liver localized HCV-specific T cell population present within the liver prior to transfection.

While this study is an important first step in showing that peripheral immunization targeting the non-structural NS4B-NS5B region of HCV can produce potent and function T cell responses within the liver, there were several limitations to our study that merit further study. First, these experiments were performed in one strain of inbred mice, additional studies are needed in order to assess whether these findings would be replicated in a population of outbred humans. Second, our study was limited in that vaccine-specific IFN-γ secretion was used as the main marker of immunogenicity. Additional studies are needed in order to assess the ability of these constructs to induce other forms of immunity including polyfunctional T cell responses. And lastly, additional longer-term memory studies will have to be undertaken to determine exactly how long these vaccine-specific T cells persist and remain functional within the liver.

In conclusion, our study has shown that DNA immunization with non-structural HCV proteins NS4B, NS5A and NS5B is able to induce potent HCV-specific T cell responses within the murine liver. Taken together, these findings may have important implications for future HCV vaccine development in humans.

## Supporting Information

Figure S1 **DNA and Protein Sequence for pConNS4B.** The consensus sequence for NS4B was generated from 174 different genotype 1a sequences. To inhibit *in vivo* activity, a C261T mutation was made to prevent polymerization by interfering with protein-protein interactions and inhibiting the formation of replication complex [20]. An N-term IgE leader sequence (red) and a C-term HA tag (green) were added following which the sequence was codon and RNA optiminized using GeneOptimizer™ (GENEART, Germany). The final consensus sequence was synthesized, sequence verified and inserted in to the clinical expression vector pVAX (Invitrogen) by GENEART (Germany).
(TIF)

Figure S2 **DNA and Protein Sequence for pConNS5A.** The consensus sequence for NS5A was generated from 259 different genotype 1b sequences. To inhibit *in vivo* activity, three mutations were made; a C59G mutation to interfere with the conserved zinc finger domain preventing viral replication [21] and; T213A and K215G, mutations that have been shown to stop binding of hVAP-A, which is important for viral replication [22]. An N-term IgE leader sequence (red) and a C-term HA tag (green) were added following which the sequence was codon and RNA optiminized using GeneOptimizer™ (GENEART, Germany). The final consensus sequence was synthesized, sequence verified and inserted in to the clinical expression vector pVAX (Invitrogen) by GENEART (Germany).
(TIF)

Figure S3 **DNA and Protein Sequence for pConNS5B.** The consensus sequence for NS5B was generated from 174 different genotype 1a sequences. To inhibit *in vivo* activity, a Y276A mutation was made which has been shown to prevent RNA polymerase activity RNA template/primer association [23]. An N-term IgE leader sequence (red) and a C-term HA tag (green) were added following which the sequence was codon and RNA optiminized using GeneOptimizer™ (GENEART, Germany). The final consensus sequence was synthesized, sequence verified and inserted in to the clinical expression vector pVAX (Invitrogen) by GENEART (Germany).
(TIF)

Table S1 **Parent Sequences for pConNS4B, pConNS5A and pConNS5B.** For pConNS4B and pConNS5B, 174 genotype 1a sequences were used to generate the final consensus sequences. For pConNS5B, 259 genotype 1b sequences were used to generate the final consensus sequences.
(XLS)

Table S2 **Stimulatory Peptides.** 15mer peptides matching the sequence of each construct and over lapping by 8 amino acids were generated across the entire length of each construct. For each construct, these peptides were pooled into several pools for ELISpot and 1 pool for Flow Cytometry. NS4B pool 1 (peptides 1–18), NS4B pool 2 (peptides 19–37), NS5A pool 1 (peptides 1–21), NS5A pool 2 (peptides 22–42) NS5A pool 3 (peptides 43–64), NS5B pool 1 (peptides 1–21) NS5B pool 2 (peptides 22–42) NS5B pool 3 (peptides 43–63) and NS5B pool 4 (peptides 64–84).
(XLS)
