# Correction: Induction of Intrahepatic HCV NS4B, NS5A and NS5B-Specific Cellular Immune Responses following Peripheral Immunization

**DOI:** 10.1371/annotation/c338fb0b-b632-487d-96dd-d3ea666e5e26

**Published:** 2013-01-22

**Authors:** Krystle A. Lang Kuhs, Roberta Toporovski, Jian Yan, Arielle A. Ginsberg, Devon J. Shedlock, David B. Weiner

Dr. Jian Yan was not included in the author byline. She should be listed as the third author and affiliated with Inovio Pharmaceuticals Inc., Blue Bell, Pennsylvania, United States of America. Her contributions are as follows: Analyzed the data, contributed reagents/materials/analysis tools, computer data base analysis of HCV constructs, phylogenetic tree studies, contribution to T cell assays performed for mapping analysis of immune profiles.

The correct citation is: Lang Kuhs KA, Toporovski R, Yan J, Ginsberg AA, Shedlock DJ (2012) Induction of Intrahepatic HCV NS4B, NS5A and NS5B-Specific Cellular Immune Responses following Peripheral Immunization. PLoS ONE 7(12): e52165. doi:10.1371/journal.pone.0052165

There was an error in the Competing Interests statement. The correct statement is: We have the following interests. The Weiner Laboratory notes collaborations and consulting with industry which in the interest of disclosure are reported here. Over the past three years DBW has received consulting fees, stock ownership, or royalties through service on Scientific Advisory Boards, Review Boards, Speaking support with the following companies: Pfizer, Inovio, BMS, VGXi, Virxsys, Ichor, Merck, Althea, Aldeveron, Novartis and possibly others. Many of these are in the DNA vaccine area. The University of Pennsylvania Conflicts of Interest committee monitors these activities. There is a patent pending on the vaccines described in the manuscript. There are no further patents, products in development or marketed products to declare. This does not alter our adherence to all the PLOS ONE policies on sharing data and materials, as detailed online in the guide for authors."

Additionally, the patent pending on the DNA constructs described in our manuscript is entitled “HCV Vaccines and Methods for Using the Same,” application number 13/127,008. 

