# Supplementary figures and images for: Induction of Intrahepatic HCV NS4B, NS5A and NS5B-Specific Cellular Immune Responses following Peripheral Immunization
Source: PLoS One. 2012 Dec 21;7(12):e52165. doi: 10.1371/journal.pone.0052165 (PMC3528776; doi:10.1371/journal.pone.0052165)

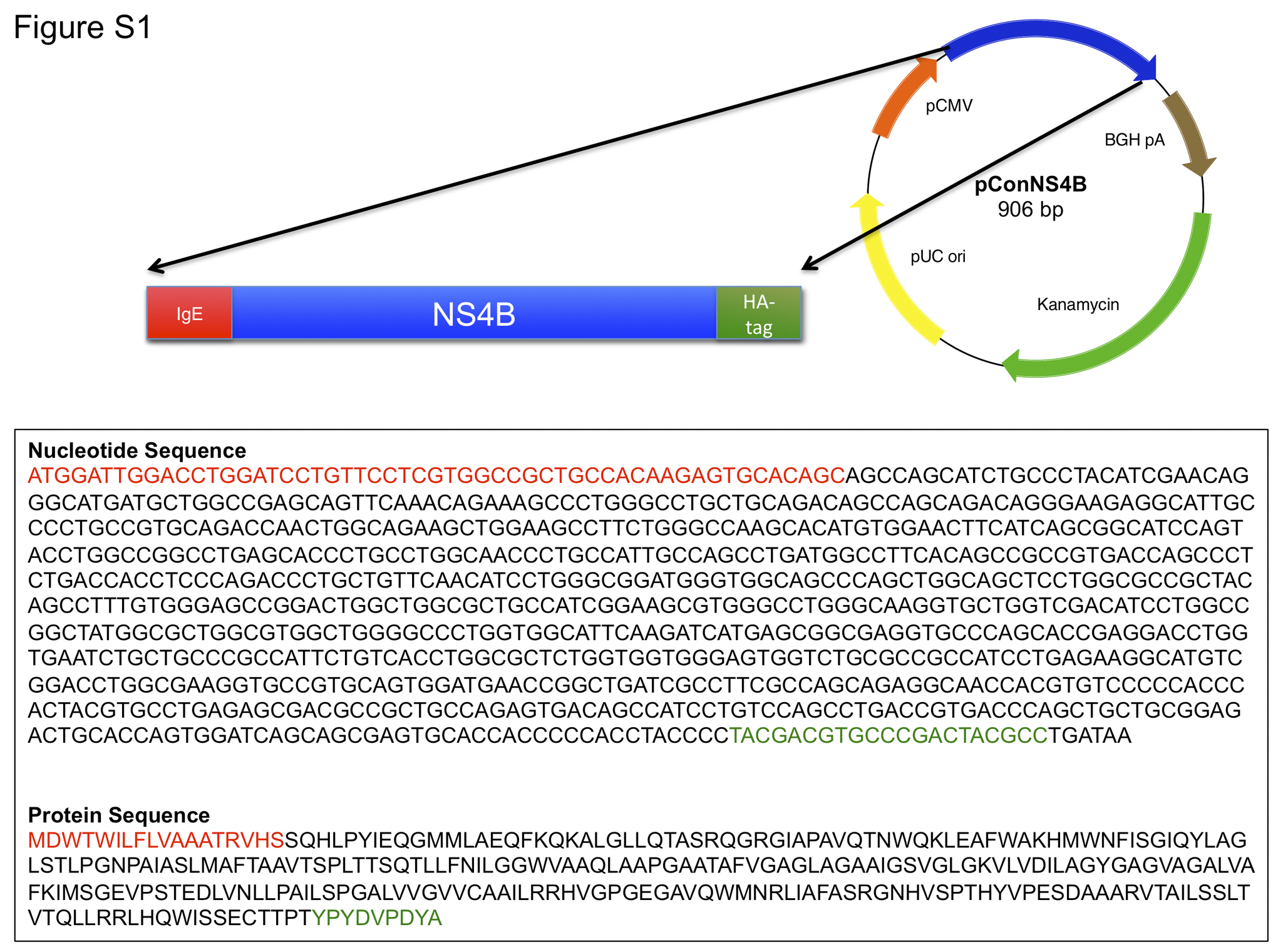

Supplement: Figure S1 — DNA and Protein Sequence for pConNS4B. The consensus sequence for NS4B was generated from 174 different genotype 1a sequences. To inhibit in vivo activity, a C261T mutation was made to prevent polymerization by interfering with protein-protein interactions and inhibiting the formation of replication complex [20]. An N-term IgE leader sequence (red) and a C-term HA tag (green) were added following which the sequence was codon and RNA optiminized using GeneOptimizer™ (GENEART, Germany). The final consensus sequence was synthesized, sequence verified and inserted in to the clinical expression vector pVAX (Invitrogen) by GENEART (Germany). (TIF) [file pone.0052165.s001.tif]

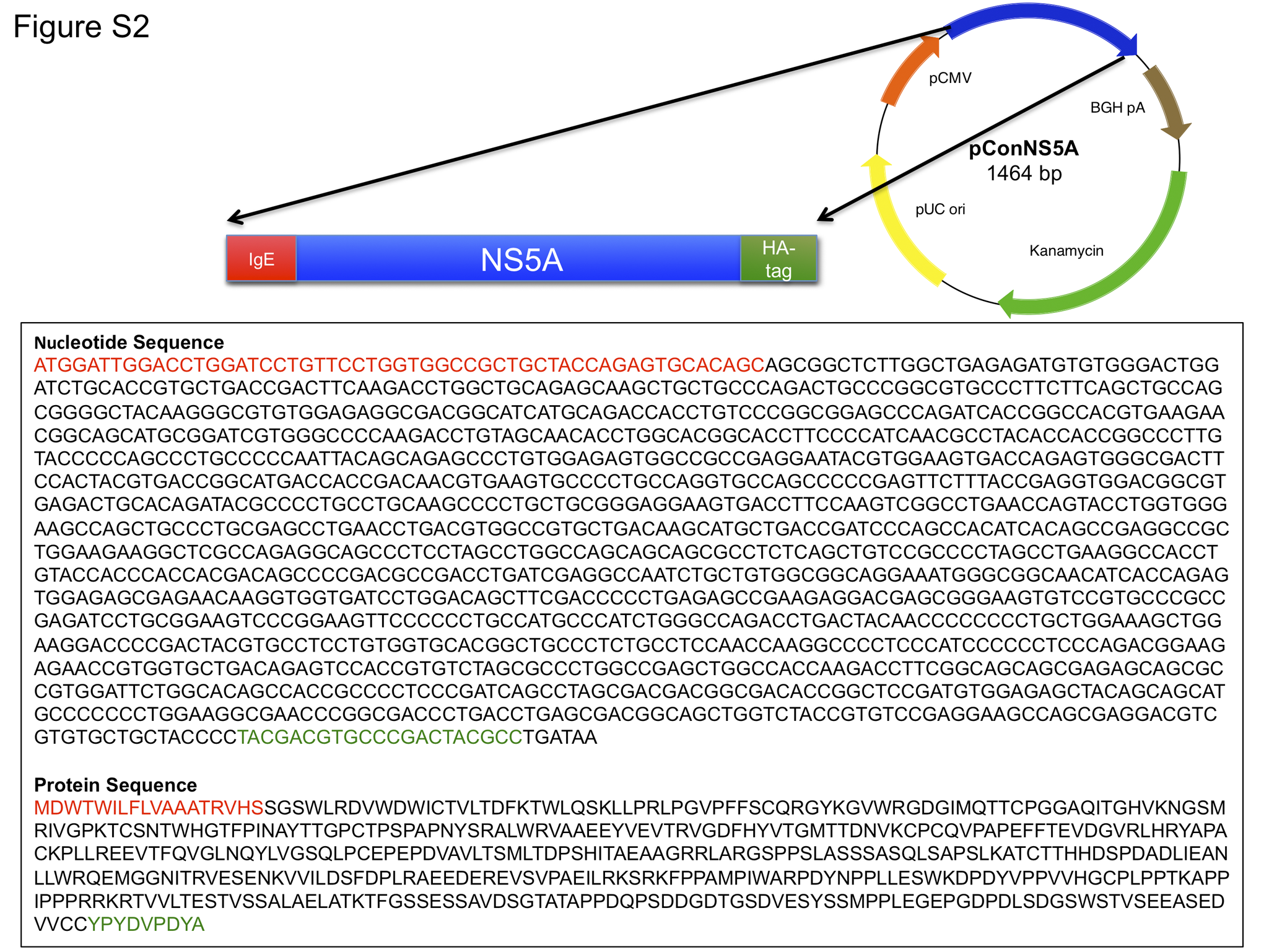

Supplement: Figure S2 — DNA and Protein Sequence for pConNS5A. The consensus sequence for NS5A was generated from 259 different genotype 1b sequences. To inhibit in vivo activity, three mutations were made; a C59G mutation to interfere with the conserved zinc finger domain preventing viral replication [21] and; T213A and K215G, mutations that have been shown to stop binding of hVAP-A, which is important for viral replication [22]. An N-term IgE leader sequence (red) and a C-term HA tag (green) were added following which the sequence was codon and RNA optiminized using GeneOptimizer™ (GENEART, Germany). The final consensus sequence was synthesized, sequence verified and inserted in to the clinical expression vector pVAX (Invitrogen) by GENEART (Germany). (TIF) [file pone.0052165.s002.tif]

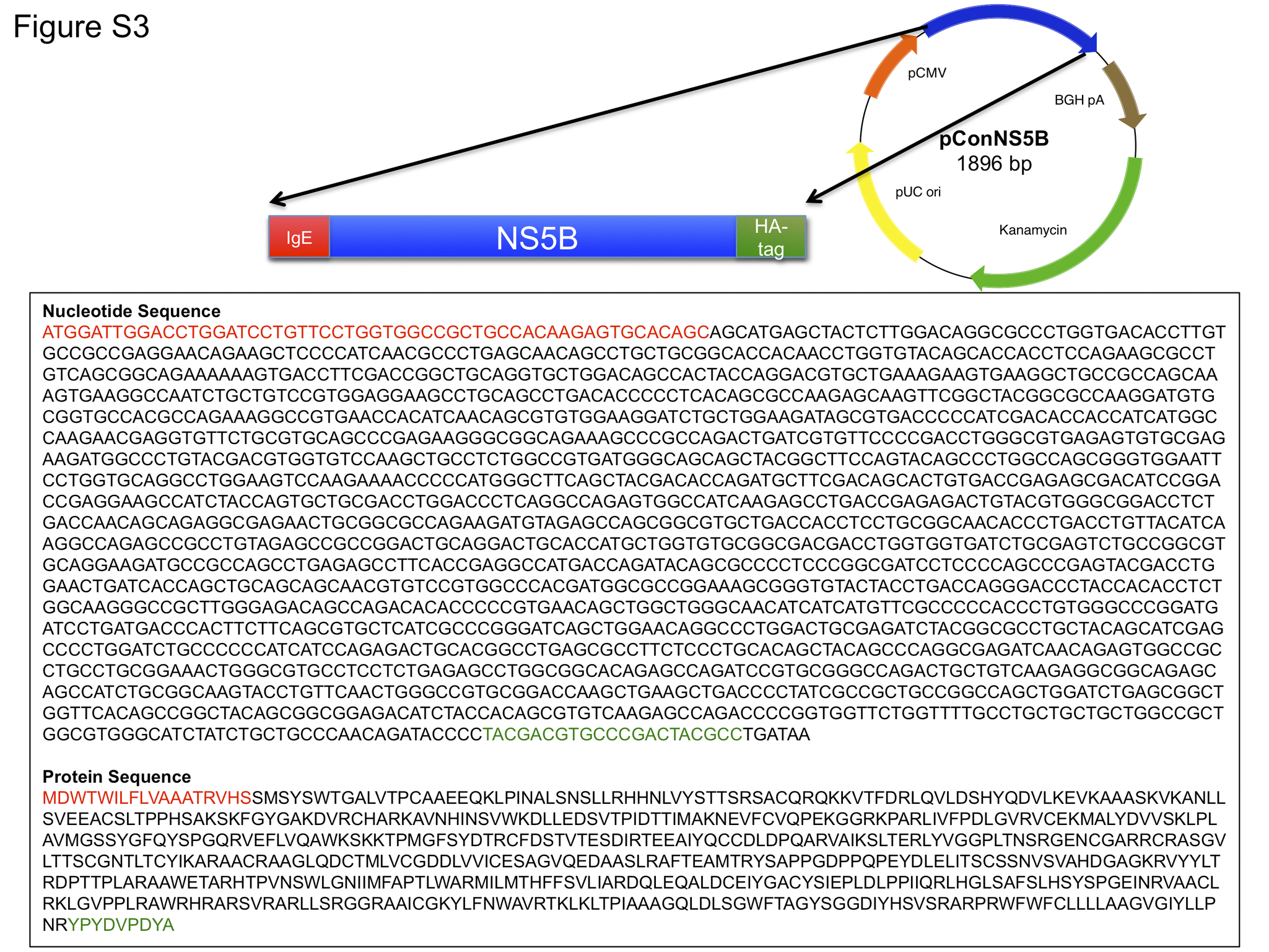

Supplement: Figure S3 — DNA and Protein Sequence for pConNS5B. The consensus sequence for NS5B was generated from 174 different genotype 1a sequences. To inhibit in vivo activity, a Y276A mutation was made which has been shown to prevent RNA polymerase activity RNA template/primer association [23]. An N-term IgE leader sequence (red) and a C-term HA tag (green) were added following which the sequence was codon and RNA optiminized using GeneOptimizer™ (GENEART, Germany). The final consensus sequence was synthesized, sequence verified and inserted in to the clinical expression vector pVAX (Invitrogen) by GENEART (Germany). (TIF) [file pone.0052165.s003.tif]
